# Building the bridge as you cross it – a process evaluation of the implementation of a clinically integrated living lab in Swedish public primary care

**DOI:** 10.1080/02813432.2026.2752760

**Published:** 2026-10-28

**Authors:** Sarah Samuelson, Eva-Lisa Petersson, Dominique Hange, Cecilia Björkelund, Sven Persson Kylén, Malin Östman, Irene Svenningsson

**Affiliations:** ^a^ General Practice/Family Medicine, School of Public Health and Community Medicine, Institute of Medicine, Sahlgrenska Academy, University of Gothenburg, Gothenburg, Sweden; ^b^ Region Västra Götaland, Research, Education, Development & Innovation (REDI), Primary Health Care, Gothenburg, Sweden

**Keywords:** Primary care, employee-driven innovation, living lab, user involvement, process evaluation, Sweden

## Abstract

**Background:**

Ongoing digital transformation is pressuring primary care to adopt new technologies, underscoring the need for clinic-based innovation arenas such as living labs and employee-driven innovation.

**Objective:**

To evaluate the implementation process of a clinically integrated living lab at a Swedish primary care centre, where employee-driven innovation is utilised to meet healthcare needs.

**Study design:**

We conducted a process evaluation of the implementation of a clinically integrated living lab guided by the Medical Research Council guidelines, focusing on context, implementation, impact mechanisms and their interrelations.

**Participants:**

Healthcare professionals; primary care managers; executive managers in innovation, research and development; and staff in innovation-supporting roles.

**Data collection and analysis:**

Qualitative data were collected over several years, including focus group interviews, dyadic interviews and various documents. All data were analysed using systematic text condensation.

**Results:**

Key findings indicate that the living lab’s clinical embeddedness, combined with strategic leadership, facilitated implementation by enabling adaptability, organisational integration and employee engagement. However, it also introduced vulnerability to internal and external influences. Main barriers to implementation included a lack of long-term financial sustainability and challenges navigating complex regulations governing public–private innovation collaborations.

**Conclusions:**

The living lab emerged as a bottom-up initiative deeply rooted in everyday healthcare practice and continuously adapting and evolving in response to its environment, embodying the metaphor of ‘building the bridge as you cross it’. The findings can inform decision-makers in building sustainable, clinically embedded innovation structures in primary care and support leaders in fostering sustained employee engagement in innovation.

## Introduction

The rapid digitalisation of healthcare is placing increasing demands on primary care to adopt and adapt to new technologies and services. This creates a need for clinic-based arenas where innovations can be systematically co-developed, tested and evaluated in real-world practice [1,2]. Living labs offer a promising approach for bridging the gap between technological development and everyday clinical work.

Living labs are commonly described as collaborative arenas where stakeholders from the public sector, industry, academia and end users jointly create, test and refine innovations in real-world contexts [3]. In healthcare, test environments are intended to improve care quality while fostering the development, testing and adoption of sustainable e-health solutions through collaboration, innovation and knowledge exchange [4]. Living labs are test environments distinguished by operating in real-world contexts and placing strong emphasis on user involvement [5,6]. As a living lab supports the development of complex solutions in dynamic settings, this approach is particularly well-suited to addressing health issues among older adults and vulnerable populations [5]. Moreover, user involvement in the innovation process increases the likelihood that solutions will be well-adapted to their intended use [7,8] and facilitates successful implementation [9]. This is especially important when a health innovation must be tailored to a specific population or context [8,10]. While previous research has examined the feasibility, adoption and implementation of specific innovations (e.g. technological solutions) [9,11–13], less attention has been paid to how living labs as an organisational concept are integrated into routine clinical practice and function as structures for innovation [14]. However, a limited number of studies have addressed this perspective [14–16].

Employee-driven innovation is another inclusive approach that draws on employees’ contextual knowledge and experience in the innovation process. It engages employees without formal innovation roles, who apply insights from their daily work to improve processes, products or services [17,18]. Employee-driven innovation can therefore be understood as a creative workplace activity that promotes both innovation and learning, as it demands new ways of responding to challenges, with ordinary employees playing a key role [19]. In this way, it can contribute to both employee empowerment and the development of collective agency – manifested as the shared capacity of individuals to act together to shape conditions, make decisions and drive change [20].

Drawing on this background, living labs and employee-driven innovation emerge as complementary approaches to innovation informed by practice, each engaging different yet overlapping forms of experiential knowledge and user participation. In this study, the living lab is conceptualised as a collaborative structure, while employee-driven innovation represents the practice-based processes through which innovation is initiated and developed by healthcare professionals. Although these approaches have been studied separately, there is a lack of research exploring their possible integration.

To our knowledge, this is the first clinically integrated living lab within a Swedish primary care centre (PCC). In the living lab, employees continuously engage in innovation alongside routine clinical work. Region Västra Götaland (VGR) plans to expand this model to multiple sites; however, there is limited knowledge of how living labs can be effectively implemented and embedded in public primary care. This study therefore seeks to generate insights to inform future implementation.

## Objective

The objective of this study was to evaluate the implementation process of a clinically integrated living lab at a Swedish PCC, where employee-driven innovation is utilised to meet healthcare needs.

## Methods

### Study design

This study evaluates the implementation of a clinically integrated living lab at a public PCC from August 2019 to December 2025. Guided by the Medical Research Council (MRC) guidelines [21,22], this empirically grounded process evaluation examined how context, implementation and impact mechanisms interacted to shape both the living lab and its implementation. More specifically, we describe

- Contextual factors that influenced, interacted with and were shaped by implementation;
- The implementation process, including the delivery, training, resources and adaptations; and
- Factors facilitating or hindering implementation.

The MRC framework is well-suited to guiding process evaluations of public health interventions and complex intervention research in health services [21]. Complex interventions are characterised by multiple interacting components, diverse stakeholder involvement and contextual adaptability [22]. Living labs share these same features [23] and thus require structured yet flexible implementation across healthcare levels. The MRC framework has previously been applied in studies involving the implementation of care manager in collaborative care [24] and the implementation of a model for a person-centred, collaborative approach for patients on sick leave due to mental health issues [25]. This manuscript was prepared in accordance with the Standards for Quality Improvement Reporting Excellence (SQUIRE) 2.0 guidelines for reporting quality improvement studies [26].

### Setting

Sweden has 21 regions and 290 municipalities, which are responsible for organising and funding healthcare. Regions manage primary and hospital care, while municipalities handle care for older people and those with disabilities. Of about 1200 primary care centres, 60% are public and 40% private, and patients can choose their provider [27].

This study was conducted in VGR, the second largest region in Sweden, with a population of approximately 1.8 million. The Innovation Platform is VGR’s regional support system for facilitating and guiding healthcare innovation. The living lab was implemented at a public PCC serving approximately 5300 inhabitants in a rural area on the west coast of Sweden. During the study period, the PCC employed 22–25 staff members, including general practitioners, resident physicians, nurses, specialist nurses, assistant nurses, a psychologist, a psychotherapist and a podiatrist.

### Outline of the living lab intervention

The living lab is clinically integrated within a public PCC, where employees engage in public–private innovation collaborations alongside their regular clinical duties. In addition, PCC staff participate weekly in employee-driven innovation processes aimed at developing and improving clinical practice, as described by Samuelson et al. [20] (Figure 1).

**Figure 1.**
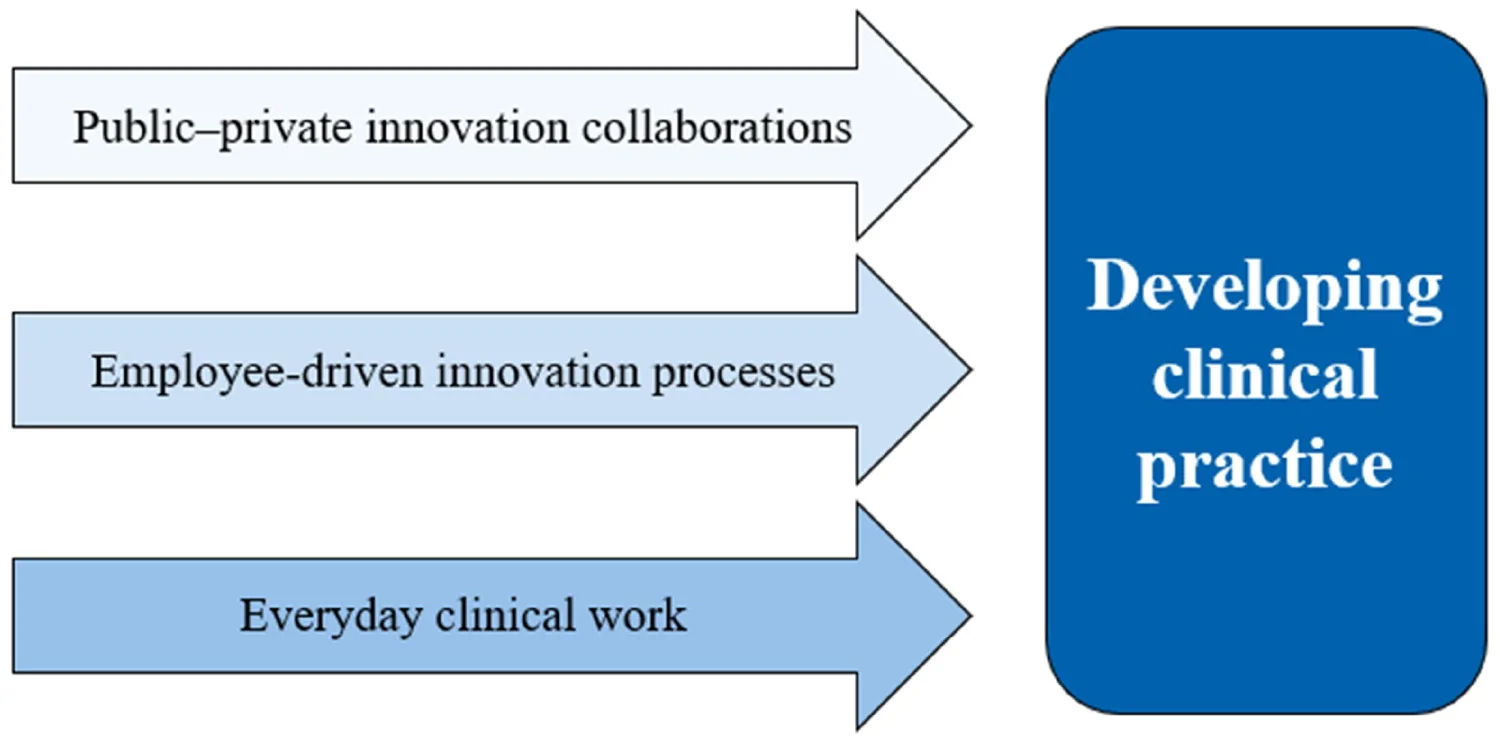
Overview of staff engagement in innovation at the PCC.

The following is a description of the living lab intervention, outlining its key roles and functions, organisational framework and processes for both external and internal projects.

#### Key roles and functions

The living lab is sustained and driven by specific key roles and functions. The project leader holds a coordinating role with the primary care organisation and the region, managing project requests and facilitating their development through liaisons with companies, the Innovation Platform, information technology (IT) services, legal services, procurement staff and other relevant stakeholders. The local project leader, who also works as a district nurse at the PCC, engages employees, communicates living lab activities to staff and oversees project implementation. Both project leaders collaborate closely on all projects. The research coordinator provides administrative support, while the PCC manager and the team leader facilitate employee participation by ensuring the necessary scheduling arrangements.

#### Organisational framework

All key roles have distinct functions, and the individuals playing these roles are part of groups with specific purposes. Together, these groups form the organisational framework of the living lab, with the key roles and functions in each group illustrated in Figure 2. All living lab activities are based on stakeholder involvement and collaboration. At the planning and implementation level, the local working group meets once a month to discuss incoming project requests as part of the needs assessment. The local planning group meets weekly to plan and allocate time for employees to participate in upcoming living lab projects. The preparatory group meets several times a week and is continuously involved in managing project processes – from needs assessment and stakeholder coordination to regulatory compliance, execution and evaluation. At the strategic level, the steering committee provides advisory support and strategic guidance and meets every other month.

**Figure 2.**
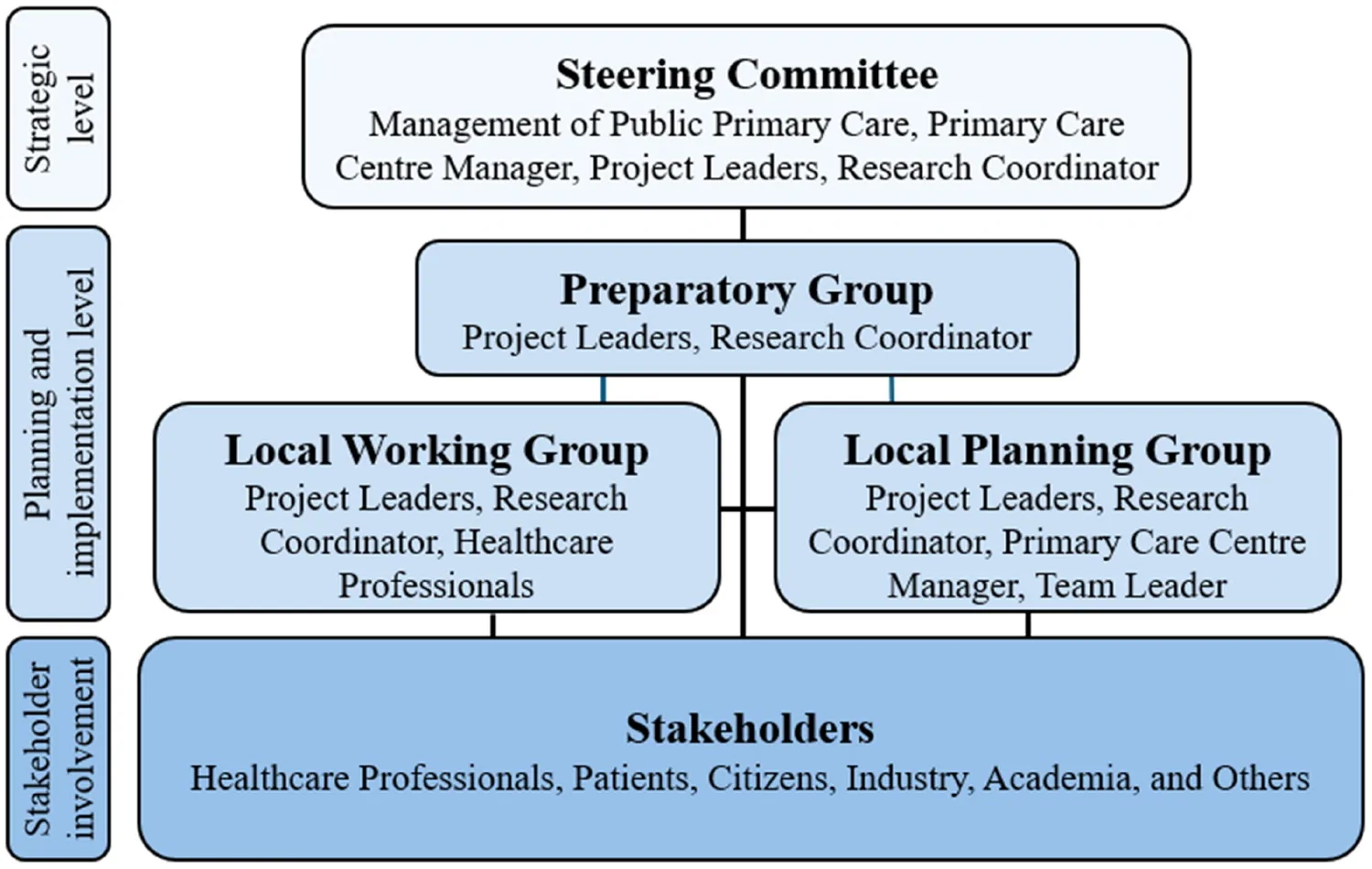
Organisational framework of the living lab, showing the different groups and the key roles and functions within each group.

#### Processes for external and internal living lab projects

The living lab receives project requests from companies (external projects) and from the primary care organisation or the region (internal projects), with examples presented in Supplementary File 1. The following describes the process by which project requests develop into full projects, starting with external projects (Figure 3) and followed by internal projects (Figure 4).

**Figure 3.**
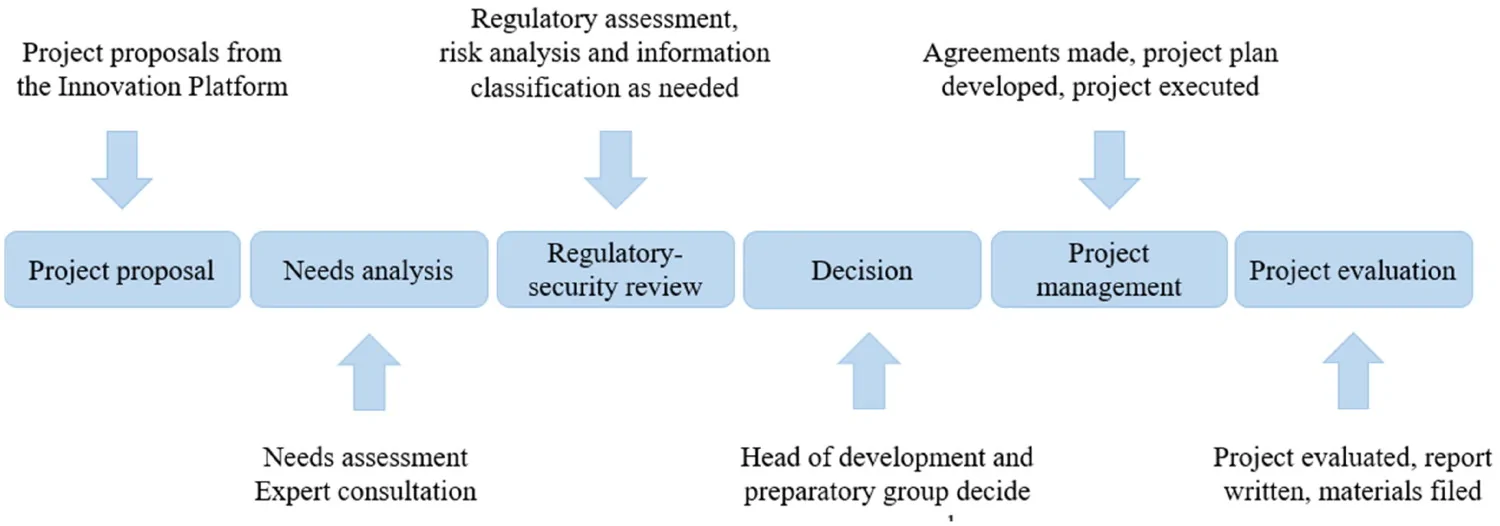
The process followed for external projects.

**Figure 4.**
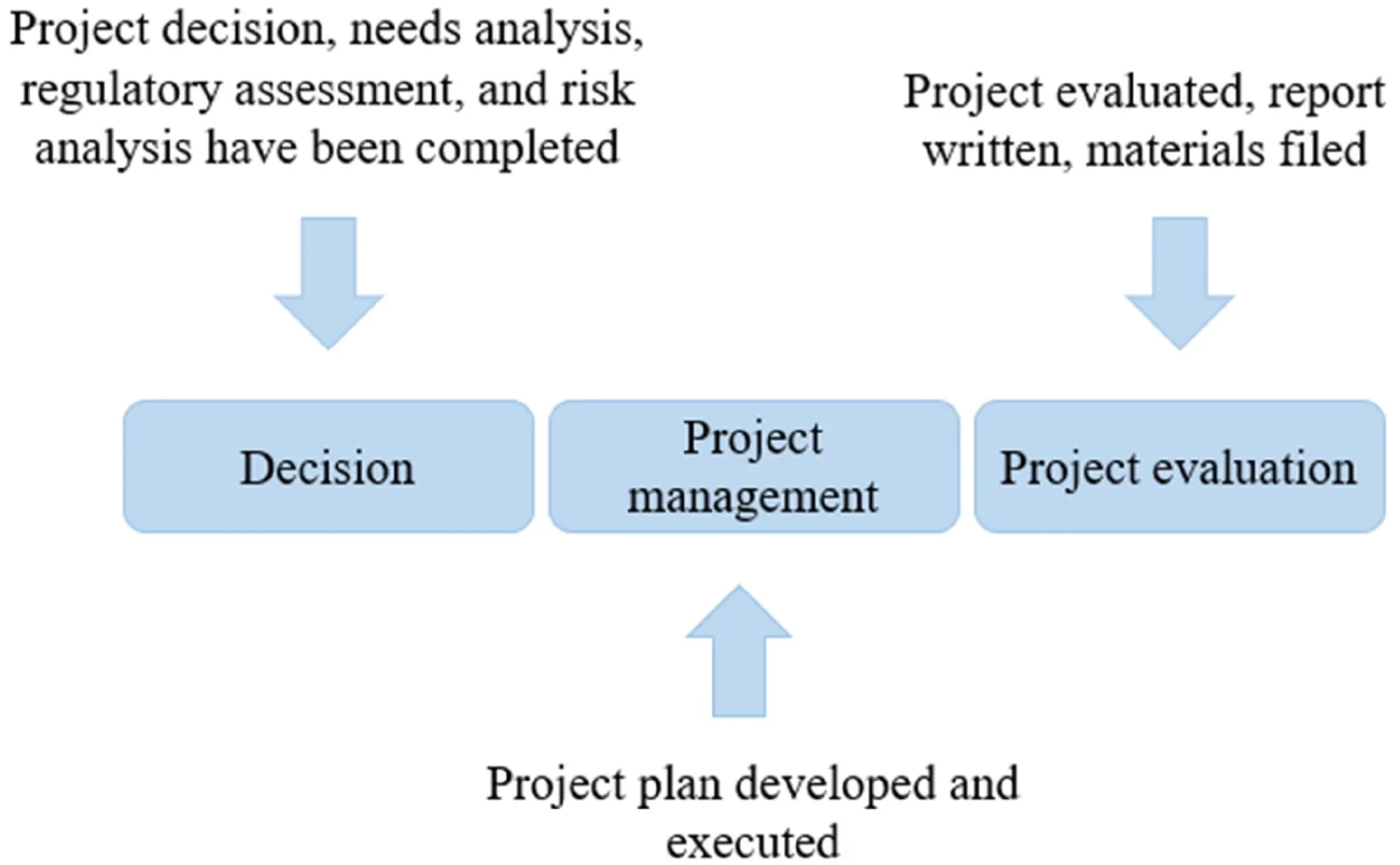
The process followed for internal projects.

##### External projects

External project requests reach the living lab via the Innovation Platform. The preparatory group conducts a multi-part needs analysis using the Need, Approach, Benefits and Competition (NABC) method [28], including regulatory assessment and an inventory of existing activities. The local working group and experts in the field are also consulted to assess clinical relevance. Based on the results of the needs analysis, the preparatory project group and the head of development decide on project approval. If approved, agreements are signed, a project plan is created and the local project leader engages healthcare professionals relevant to the specific project and oversees its execution. The project concludes with a final report and archiving of materials. The external project process is illustrated in Figure 3.

##### Internal projects

Internal projects are initiated by the region or the public primary care organisation. These projects often involve goods and services that have already been procured and need to be tested on a small scale before broader implementation. As a result, the needs analysis, overall security assessment and decision to proceed are made before the project reaches the living lab. Thus, the next step involves developing a project plan, engaging healthcare professionals as appropriate and executing the project. Upon completion, an evaluation is conducted, a final report is prepared and project materials are archived. The internal project process is illustrated in Figure 4.

### Data collection

The process evaluation is based on extensive data collected between 2019 and 2025, as detailed in Tables 1–3. Dyadic and focus group (FG) interviews previously conducted for earlier publications were re-analysed after obtaining consent from all participants. For further details on data collection and sampling, see the original publications [7,20]. The data also included various documents, such as notes from steering, working and planning group meetings; project reports; minutes; and field notes. To further enrich the data and broaden the perspective, two additional dyadic interviews were conducted in November 2023. One interview included two healthcare professionals with extensive experience from the local working group, stakeholder meetings and multiple living lab projects, while the other included the PCC manager and the team leader. These interviews were conducted at the PCC during working hours by the first (SS) and last (IS) authors. SS led the interviews, while IS observed and asked supplementary questions as needed. The questions addressed key areas such as experiences and perceptions of participating in living lab activities, individual and organisational contributions, and future perspectives. The interviews lasted 63 and 58 min, respectively.

**Table 1.** An overview of data sources, participants and time of execution.

Data source	Participants	Gender	Age (years) range/mean/median	Time of execution
FG 1	4 executive managers in the fields of innovation, research and development	2 females, 2 males	40–64/54/57	September 2024
FG 2	4 individuals in regional innovation-supporting roles (i.e. IT, procurement, clinical research, innovation platform)	2 females, 2 males	41–57/51/53	September 2024
FG 3	3 primary care managers at different organisational levels, 1 research coordinator	3 females, 1 male	51–61/55/54	September 2024
FG 4	5 healthcare professionals: 1 resident physician, 1 nurse, 1 assistant nurse, 1 medical secretary, 1 psychotherapist	4 females, 1 male	25–59/42/42	November 2024
FG 5	5 healthcare professionals: 1 resident physician, 2 district nurses, 1 assistant nurse, 1 medical secretary	4 females, 1 male	41–65/50/49	November 2024
Dyadic interview 1	2 nurses/district nurses	2 females	47–53/50/50	November 2023
Dyadic interview 2	1 PCC manager, 1 team leader	1 female, 1 male	50–60/55/55	November 2023
FGs 1–3 from [20], re-analysis	16 healthcare professionals: 1 general practitioner, 3 resident physicians, 7 district nurses/nurses, 1 assistant nurse, 3 medical secretaries, 1 psychotherapist	13 females, 3 males	23–59/45/44	May 2021
Dyadic interviews 1–9 from [7], re-analysis	1 nurse, 1 occupational therapist, 2 entrepreneurs, 2 older people	3 females, 3 males	32–79/51/42	December 2020–June 2021
Documents	Minutes from steering group, local working group and planning group meetings; project reports; field notes			2019–2025

**Table 2.** Participants with repeated interviews and number of interview sessions.

Participants	Number of interviews	Interview sessions
1 nurse	4	3 dyadic interviews from [7] FG 1 from [20]
2 entrepreneurs 2 older people 1 occupational therapist	3	3 dyadic interviews from [7]
1 district nurse 1 nurse	3	FGs 2 and 3 from [20] Dyadic interview 1 FG 5
1 PCC manager 1 team leader	2	Dyadic interview 2 FG 3
1 resident physician 1 medical secretary 1 psychotherapist	2	FGs 1 and 3 from [20] FG 4

**Table 3.** Overview of the datasets employed in the key components of the process evaluation framework.

Key component	Description	Data sources
Context	Contextual factors that influence or are influenced by the implementation of the living lab	FGs 1–5 Dyadic interviews 1–2 Documents FGs 1–3 from [20], re-analysis
Implementation	Components of the living lab and mode of delivery	Dyadic interview 1 Documents Dyadic interviews 1–9 from [7], re-analysis
Mechanism of impact	Responses to and interactions with the living lab	FGs 1–5 Dyadic interviews 1–2 Documents FGs 2–3 from [20], re-analysis Dyadic interviews 1–9 from [7], re-analysis

In autumn 2024, five additional FG interviews were conducted with executive managers in the region (FG 1), regional innovation-supporting staff (FG 2), primary care managers at different organisational levels and a research coordinator (FG 3) and healthcare professionals (FGs 4–5). FGs 1–3 were held digitally and FGs 4–5 were held at the PCC, all during working hours. The interviews were conducted by the first (SS, FGs 1–3), second (E-LP, FGs 1–4), third (DH, FG 5) and last (IS, FGs 4–5) authors. SS and IS served as moderators, while E-LP and DH participated as note-taking observers. To facilitate the discussion, the moderator used a semi-structured interview guide with open-ended questions, which is provided in Supplementary File 2. Each interview lasted approximately 50 min.

A total of 38 individuals participated in the study interviews. Thirteen were interviewed more than once, resulting in 60 interview participations in total (Tables 1 and 2).

### Data analysis

All data were analysed using systematic text condensation (STC), as described by Malterud [29]. As a qualitative, inductive and data-driven method, STC was chosen to ensure a manifest, descriptive analysis rather than an exploration of potential underlying meanings [29]. The focus group and dyadic interview formats allowed participants to respond to and build on each other’s experiences. The analysis was conducted by SS, E-LP and IS. SS and IS were involved in the development of the living lab from the outset, while E-LP had no prior experience with the living lab. Preconceptions were addressed through reflexivity and ongoing discussions throughout the analysis [30].

The analysis was conducted as a systematic, iterative and flexible process in four steps. First, to gain a comprehensive understanding of the data, all transcriptions were read multiple times by two authors (SS, E-LP). Second, these two authors independently coded the transcripts by identifying and labelling meaning units, guided by the study’s aim. In this step, SS brought an insider perspective while E-LP contributed an outsider perspective, facilitating critical reflection and discussion during the coding process. The primary analyst, SS, then organised the codes into code groups and subsequently into subcodes following in-depth discussions with E-LP and IS. Third, the meaning units in each subcode were reduced into a condensate. Fourth, descriptions were generated to reflect key components of the implementation process. Throughout the analysis, ongoing discussions were held among the three authors (SS, E-LP and IS) to ensure a transparent and trustworthy analysis [30]. The study aim guided the interpretation, and both confirming and disconfirming accounts were treated equally and incorporated into the analysis. To further enhance credibility, codes were kept close to the original descriptions and discussed until consensus was reached among all authors.

## Results

The results are presented in accordance with the MRC framework [21] and cover contextual factors, the implementation process, and mechanisms of impact (expressed as facilitating factors and barriers).

### Contextual factors

The analysis of the field notes and FG interviews revealed that primary care is a resource-strained organisation, leaving little scope for conducting innovation, research and other non-clinical or non-reimbursable activities. High workloads and staff shortages were common features of everyday clinical practice. The analysis also indicated that innovation in healthcare is a recognised priority within VGR. Although VGR lacks a formal innovation strategy, it supports innovation through initiatives such as the Innovation Platform. Consequently, the living lab emerged within an environment that was both challenging and supportive.

The living lab was developed in a PCC with a history of working with innovation, where weekly time was set aside for employee-driven initiatives alongside involvement in the living lab. This environment fostered collaboration and psychological safety. Over time, it also enhanced the involved healthcare professionals’ reflective, critical and problem-solving abilities, which proved valuable in living lab projects that often required flexible and innovative thinking.

### Implementation

The living lab began as a grassroots initiative; it built on the PCC’s innovation journey, which started in early 2016 with the integration of employee-driven innovation into weekly routines. Over time, ideas for collaborating with external stakeholders to contribute to health innovation in primary care emerged. Through a 3-year EU-funded Interreg project (VITAL), the clinically integrated living lab began to take shape in autumn 2019. In 2023, the regional public primary care organisation launched a 3-year strategic initiative to govern the living lab. Drawing on the insights gained from the implementation, the regional public primary care organisation planned in 2025 to scale up the initiative, planning the establishment of a living lab network throughout the region (Figure 5).

**Figure 5.**
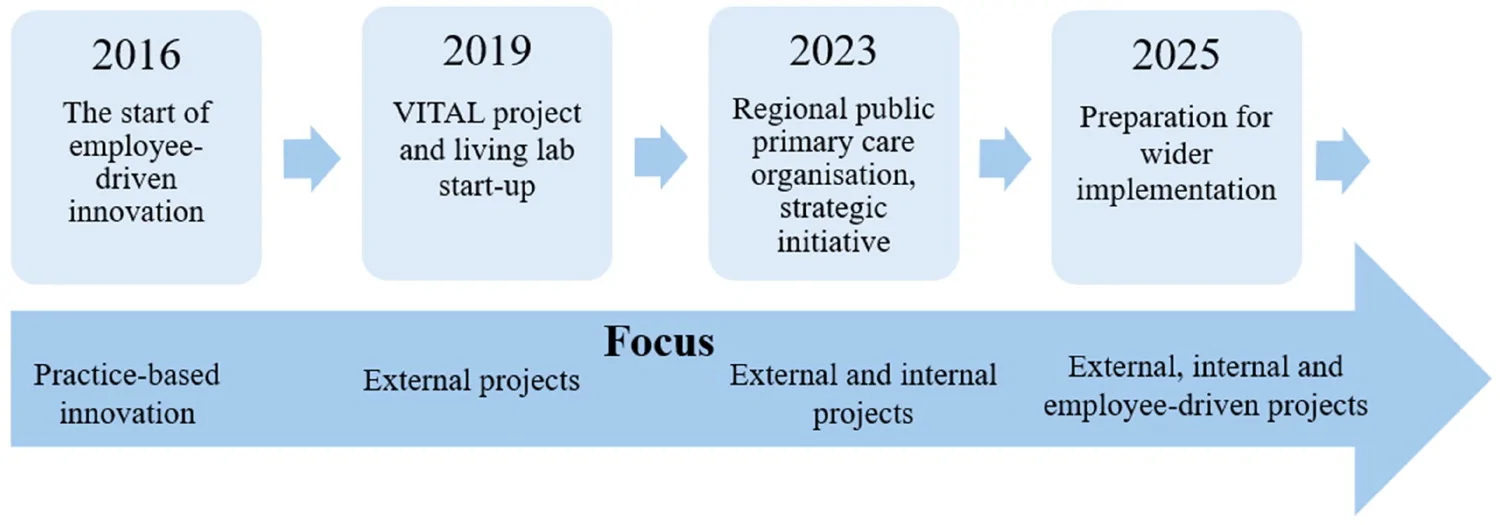
The living lab timeline.

Since its inception, the living lab has gradually evolved, shaped by stakeholder input, regulatory considerations and strategic objectives. Adaptability was therefore a key aspect of its implementation. For example, project proposals initially originated mainly from external companies; however, following the transfer of governance to the regional public primary care organisation, the focus shifted towards strategically aligned internal initiatives and subsequently towards employee-driven projects as well. Likewise, key roles, organisational structures and processes have emerged over time as a result of stakeholder feedback.

The project leader and the local project leader, with their distinct yet complementary roles, emerged as key drivers of the implementation process. Their collaboration and dual anchoring – at the regional/system level and the local level, respectively – supported integration across organisational levels. Early in the process, employee engagement was recognised as essential for reinforcing the bottom-up approach and local anchoring. A local working group, including two staff representatives, was therefore established at the PCC and met monthly to review and assess incoming project proposals. In response to feedback and to further increase employee engagement, these monthly meetings were opened to the entire staff starting early in 2025. Over time, this broader participation shifted the meetings into a space for collective reflection and gave rise to several employee-driven projects addressing identified care needs.

Another important aspect of the implementation process was the need for ongoing coordination between living lab projects and clinical operations, as participation required temporary release from clinical duties. To manage this issue, a planning group was established that met weekly to coordinate employee scheduling for the projects. When the governance of the living lab was transferred to the public primary care organisation, a steering group was formed to strengthen strategic alignment, foster organisational integration and increase resource efficiency through internal collaboration.

Early living lab projects revealed the need for structured processes for project preparation, implementation and evaluation. Several aspects of these processes – such as needs analyses, risk assessments, agreements and ethics – required consideration prior to project initiation. Project participants also requested clear project descriptions. Consequently, templates, checklists and clear processes were created to provide structure and guidance for each project.

Resources were allocated to the living lab on an annual basis to support its viability. Funding equivalent to a district nursing position was used to strengthen management and staffing, enabling employee participation in projects. Several employees also attended two service design courses to better understand user needs and engage in co-creation. When governance shifted to the public primary care organisation, the living lab also gained access to administrative support.

### Mechanisms of impact

The data analysis identified six facilitating factors and five barriers, which are presented in Table 4. These are described below with illustrative quotes.

**Table 4.** Facilitating factors and barriers.

Facilitating factors	Barriers
A need for living labs in primary care	Lack of a sustainable financing model and long-term strategy
Innovation-supporting functions and structures	Obstacles to promotion and knowledge transfer
Embeddedness in clinical practice, supported by strategic leadership	Resource-strapped primary care
Resilient and trust-based leadership	Complex and ambiguous regulatory aspects
Promotion of employee engagement in innovation	Diverging needs across organisational levels
Enhanced collaboration, competence and knowledge sharing in innovation	

#### Facilitating factors

##### A need for living labs in primary care

Participants recognised the potential for living labs in primary care. They emphasised that, in order to advance healthcare and remain relevant, primary care must innovate in collaboration with industry and other stakeholders.

For me, it feels like this type of living lab, where healthcare and industry collaborate in a sustainable and mutually beneficial way, is crucial for us to remain relevant in the future as publicly funded healthcare. (FG 1)

Moreover, the participants highlighted the need for primary care to be the driver of its own development. They saw living labs as a way for primary care to identify its needs in both care processes and technology, enabling active participation in strategic planning rather than merely responding to external demands.

There is far too much … well … being decided by those who don’t work in primary care on behalf of primary care, if I may put it that way. I believe primary care itself needs to take charge of this. (FG 3)

While company inquiries to the living lab varied with market conditions, interest remained strong in testing solutions in a real clinical setting with healthcare professionals and end users. Companies gained valuable insights into product performance in the intended environment, leveraged user input for refinement and fostered developer engagement through direct feedback.

##### Innovation-supporting functions and structures

The participants acknowledged that driving innovation in healthcare requires a broad range of specialised knowledge. Thus, being part of a large healthcare organisation with access to expertise in areas such as legal matters, IT, research and innovation processes was a key factor.

… if we had not accessed the expertise we needed, I don’t think we would have had the strength to remain persistent. (FG 1)

The project leader served as a liaison between the living lab and its stakeholders, described as the ‘spider in the web’, fostering collaboration and developing and maintaining innovation-supporting networks tailored to the living lab. With the support of diverse expert competencies, the project leaders carefully prepared each project to ensure compliance with applicable laws and regulations. This provided a sense of security for both the primary care manager and the healthcare staff responsible for conducting the tests.

It’s a really extensive job that is done first … which also makes you feel confident that what we are going to test here, as you said, is legally okay […] and that’s also the reassurance of being part of a large organisation because then you have access to this knowledge. (FG 3)

According to participants, the presence of an innovation-supportive culture, along with employees’ knowledge and hands-on experience in innovation processes, was a key prerequisite for implementing a clinically embedded living lab. In this way, the weekly employee-driven innovation activities provided an important foundation for the further development of the living lab.

There must be certain basic prerequisites to make this possible. One of these prerequisites is that innovation or improvement work needs to be considered in the workplace before taking the step towards something larger. (FG 3)

##### Embeddedness in clinical practice, supported by strategic leadership

Embedding the living lab within clinical practice, as opposed to operating the lab as a separate test unit outside the clinic, was described as a facilitating factor. The embedded structure enabled the living lab to adapt more easily to internal and external changes.

It also proved advantageous, especially in terms of resource management. When living lab activities slowed down, focus could be redirected to clinical tasks to maintain productivity.

I believe that [the clinical embeddedness] is the real golden opportunity that should be truly valued. In my opinion, it has been a truly significant success. (FG 1)

As a clinically embedded unit, it was important for the living lab to work closely with primary care leadership to avoid becoming isolated within the broader organisation. This ensured alignment with strategic priorities, optimised coordination and leveraged existing resources and knowledge. Clinical embedding also facilitated the seamless integration of innovation into daily practice and fostered employee engagement.

It’s extremely important that the leadership [within the public primary care organisation] has a deep understanding, awareness and commitment […] so that you don’t end up as an isolated entity, a satellite floating around, unknown to your leadership, because that makes it difficult to achieve the coordination effects – and, as [name] said, balancing production resources with the test resources. (FG 1)

I don’t usually think of this as my assignment at the primary care centre or as part of the living lab, but in a way, they are so closely intertwined. (FG 4)

##### Resilient and trust-based leadership

Running a clinically integrated living lab was often resource-intensive and demanding, requiring the leadership to demonstrate resilience and persistence. In addition to having an interest in innovation, participants emphasised the importance of the manager having formal knowledge and experience in the field to ensure that innovation remained a priority alongside clinical duties, even in challenging times.

There are ups and downs, and you have to be able to handle the pressure and stress that comes with it – to manage both the highs and the lows. (FG 3)

Unfortunately, it’s not a competence that just comes from being interested – it requires a lot of reading […] I believe that *formal* competence is important. (FG 3)

Given the dynamic nature of the living lab, with activities that fluctuate over time, active and flexible scheduling was essential to maintain a balanced workload. The primary care manager’s main responsibility was to enable employee participation by allocating appropriate resources, particularly time. In turn, employees were expected to inform the manager when stress levels became excessive, while the manager remained attentive and responsive to their needs. To harness the full potential of each individual, the primary care manager refrained from micromanaging, instead recognising and trusting employees’ competence and skills.

It’s also good that he [the manager] doesn’t try to micromanage, because he doesn’t know all the pieces. That’s impossible. […] I feel like he often says, ‘Do that and show me later, because you know this best’. (FG 2 from [20], re-analysis)

It has a lot to do with trust. Trusting your employees, that they can solve this. I don’t need to solve it for them. (Dyadic interview 2)

##### Promoting employee engagement in innovation

As the living lab was built on employees’ professional and contextual expertise, their active participation was crucial from the start in order to support motivation, engagement and long-term sustainability. The role of the local project leader was viewed as central in promoting employee engagement by providing regular updates, encouraging participation and supporting project implementation.

I think it’s really important to have someone who’s constantly promoting, marketing and selling the idea … (FG 4)

Thus, employee engagement in innovation work did not occur automatically but required ongoing effort, facilitated by shared reflection. Reflection on innovation efforts enabled employees to gain new perspectives and a clearer understanding of the work’s value, forming a shared foundation for further development.

I think it’s important to have a shared platform, where most people recognise that something can be done, that change is possible and that it can simplify our daily work. If we see that, then everything becomes much easier than if half the group fails to see the benefits – whether for patients or staff. (FG 3 from [20], re-analysis)

##### Enhanced collaboration, competence and knowledge sharing in innovation

The development of the living lab revealed several innovation-related challenges, which led to the involvement of experts from different parts of the primary care organisation. The exchange of knowledge and competence flowed in both directions, with the living lab drawing on expertise from various regional support functions and others leveraging its primary care experience. Participants reported that this contributed to more efficient innovation processes, with shorter lead times, less strain on expert resources and reduced risks related to patient safety, branding, medicine and information security.

The more people who have knowledge, the better. […] instead of having to go from function to function, much of the fundamental expertise is embedded in the living lab. You can handle many of the questions on your own and call in experts when needed, rather than wasting a lot of time running around the system with your questions – leading to longer project timelines and perhaps even having to shut down projects because they can’t move forward. (FG 2)

#### Barriers

##### Lack of a sustainable financing model and long-term strategy

Several participants identified the development of a sustainable funding model as a major challenge for ensuring the long-term viability of the living lab. Over time, it became clear that the living lab’s operational costs could not be fully covered by external projects in which companies paid for services. Moreover, estimating the financial returns of internal projects proved challenging, as these primarily focused on introducing new working methods for procured products and services. As a result, the living lab relied on internal funding, provided annually through strategic funds.

I think it’s difficult to fully achieve financial sustainability using a project-based format, meaning having companies completely fund the living lab. There must be some internal resources to keep such an operation going over time. (FG 2)

##### Obstacles to promotion and knowledge transfer

The living lab concept was described as a new phenomenon in primary care that was not widely understood, which made it difficult to explain its purpose, function and appropriate applications to others. Uncertainty regarding financial sustainability also constrained long-term planning and communication initiatives (e.g. a website and outreach activities). Therefore, despite operating for several years, the living lab remained relatively unknown both within and beyond the region.

When I didn’t understand it [the living lab] myself at first, how can others be expected to understand? You need to be engaged to truly grasp what it’s about. (FG 5)

The lack of established pathways for disseminating the results from internal projects limited their broader benefits and impact within the organisation. Participants noted that test results from a small, rural PCC were sometimes dismissed by others, as different patient flows and demographics influence how users engage with a given innovation. Several participating employees also emphasised that uncertainty about whether their feedback would be utilised and implemented led to feelings of demotivation.

##### Resource-strapped primary care

A commonly shared view among participants was that achieving innovation within the context of primary care posed significant challenges. The participants described a common perception that employees were expected to manage innovation and development work alongside their clinical duties and that the time and resources required were generally underestimated.

There is a general underestimation of the resources needed to drive development and innovation. It just gets squeezed into people’s already-packed calendars. (FG 1)

Time pressure was prevalent across multiple organisational levels. Although employees expressed interest in and engagement with innovation, participating in living lab projects was sometimes perceived as an additional burden in their already-demanding work schedules. Thus, despite the resources allocated to the living lab, maintaining a balanced workload in the task-intensive environment of the PCC remained a constant challenge, particularly due to prolonged understaffing.

On paper, we have the funding of a district nurse, but, in reality, we don’t, because we’re … how many short? I can’t even count right now. So, in practice, we don’t have an extra. (FG 5)

##### Complex and ambiguous regulatory aspects

An analysis of documents revealed ongoing discussions concerning various regulatory, legal and data security issues throughout the implementation process. Key areas of concern included the General Data Protection Regulation (GDPR), procurement, patient involvement and ethical considerations. The absence of overarching national guidance on innovation-related regulatory issues left room for interpretation by regions and experts. As a result, regulatory considerations proved time-consuming and required input from multiple experts, reflecting the complexity of a legal framework with few clear-cut answers. Many projects required leadership with sensitivity and sound judgment to navigate the balance between regulatory compliance and practical feasibility. Some projects could not be carried out, often due to regulations related to the GDPR, while others were delayed, as companies needed to make adjustments to meet regulatory requirements. Moreover, patient involvement was subject to strict regulatory requirements, as only CE-marked products can be tested on patients, and then only within clinical studies or in non-commercial testing setups initiated by the primary care organisation. This limited the opportunities for patient involvement. A handbook was developed to clarify relevant regulations for living lab activities in public healthcare, serving as a practical guide.

##### Diverging needs across organisational levels

The living lab was guided by needs that were identified in primary care, in both internal and external projects. However, perceived needs varied across organisational levels, with employees focusing on patient- and practice-related concerns, while management prioritised strategically defined needs. Participants noted that such tensions between frontline staff and management were not unique to the living lab but were common throughout the primary care organisation. Instead, the living lab was viewed as a forum for addressing and discussing various – and sometimes conflicting – needs, allowing its participants to gain a broader perspective.

We have to try to address both sets of needs at the same time, and sometimes they conflict. When that happens, we need to assess the situation, plan accordingly and see if there’s a third way forward, or how to balance them effectively. But no, I actually see the living lab as an advantage. It brings these needs together and puts them on the table. (FG 1)

External projects primarily focused on developing, testing and refining products and services, while internal projects centred on implementing new workflows aligned with new technologies. While external projects were generally perceived positively by employees, due to their responsiveness to clinical needs and adaptability based on feedback, internal projects were more often described as burdensome. These projects typically involved pre-purchased products or services with limited possibilities for modification and were not always perceived as aligned with actual clinical needs, which some employees found demotivating.

… the external [stakeholders] are eager for feedback and are quick to go back, refine their product and adjust their prototype or whatever they want to improve. But I don’t experience that with [the public primary care organisation], as decisions are usually already made … and it’s [the product] pretty much already fully developed. (FG 5)

## Discussion

### Main findings

The aim of this study was to evaluate the implementation process of a clinically integrated living lab at a Swedish PCC, where employee-driven innovation is utilised to meet healthcare needs. The key findings indicate that the clinical integration of the living lab, together with strong and visible strategic leadership, was perceived as a crucial enabler of well-coordinated implementation. However, a major barrier to long-term sustainability was the absence of stable, ongoing funding, which forced stakeholders to prioritise short-term survival over strategic, long-term development. The results highlight the difficulties of navigating and interpreting the complex regulatory frameworks that govern public–private innovation partnerships. It was also found that employees’ motivation to engage in the living lab’s operations was influenced by divergent – and sometimes conflicting – needs across organisational levels.

### Discussion of the results and a comparison with existing literature

The embeddedness of the living lab offered a resource-efficient approach that supported user involvement and captured needs experienced in clinical practice. It also allowed for continuous feedback loops and short decision-making pathways, which made it possible for the living lab to evolve in response to emerging needs. These findings align with previous research showing that innovation projects that are integrated into everyday practice are easier to implement and sustain than separate, predefined and time-limited initiatives [31–33]. However, while the living lab’s clinical embeddedness facilitated organisational integration and employee engagement, it also exposed the living lab to internal and external vulnerabilities. Because of the high workload and staff shortages at the PCC, employees sometimes experienced living lab projects as an added burden in an already strained work schedule, despite having a strong commitment to innovation. Additionally, scepticism about transferring results from a small, rural setting (‘not invented here’ syndrome [34]) highlighted the need for testing in varied primary care contexts. This finding supports the idea of establishing an innovation network [35] of clinically embedded living labs across PCCs of varying size, location and patient populations, which could increase the generalisability and transferability of outcomes.

One of the key challenges in the implementation process was ensuring long-term financial sustainability. Bottom-up initiatives such as the living lab often lack robust clinical and cost-effectiveness evidence, making it necessary to approach stakeholders with alternative value perspectives to secure funding. This is particularly true in complex organisational contexts such as primary care, where a broader range of stakeholders must be considered [33]. Although demonstrating cost-effectiveness was challenging, the value of the living lab was recognised within the primary care organisation. Still, due to limited resources, the primary care organisation had to carefully prioritise its investments, leaving little room for long-term financial commitments. This affected the organisation’s ability to promote the living lab and integrate it into the organisation’s existing innovation structure for long-term development. Consistent with previous research [36,37], our findings support the view that healthcare requires a political mandate with a defined mission to engage in innovation, backed by dedicated financial resources. Moreover, earmarked innovation funding is important to ensure sustainability and to prevent such resources from being used to offset operational budget deficits [38].

This study’s findings underscore the challenges of navigating and interpreting the complex regulations that govern public–private innovation collaboration, which significantly prolonged the implementation process. Sweden’s decentralised healthcare system and weak national coordination have led to regional variation in managing digitalisation and regulations [39]. In VGR, a lack of consensus on key regulatory issues in the living lab context – particularly regarding the GDPR, ethics and patient involvement – posed challenges for clinic-level innovation. However, the living lab built regulatory expertise over time, which suggests that clinically embedded expert hubs can support regulatory compliance and foster collaboration to strengthen local innovation capacity.

The living lab was intended to address primary care needs through innovation, but perceptions of those needs varied across organisational levels. Employees found externally driven projects meaningful due to their involvement in assessing clinical relevance, while internal top-down initiatives were viewed as less engaging and burdensome. These differences reflect conflicting organisational logics, where efficiency-focused managerial logics clash with professional logics centred on care quality [40]. Thus, supporting employee motivation and engagement in internal top-down innovation initiatives requires an acknowledgement of co-existing logics and conflicting needs, along with responsive leadership that clearly connects decisions to clinical relevance and patient value. Moreover, clinic-level innovation requires adaptive, trust-based leadership that communicates a clear vision and fosters participation and shared ownership [41,42]. In this study, the healthcare professionals’ experiences in employee-driven innovation increased their problem-solving capabilities, which proved to be an asset in the living lab’s activities. This finding underscores the importance of developing local innovation capacity and engagement prior to living lab integration. Also, as employees gain innovation expertise, leadership must evolve accordingly through ongoing learning in the field of innovation.

During the course of the study (2019–2025), increasingly evident demographic changes and pandemic-driven digitalisation in primary care highlighted the need to test and implement new solutions in practice. In turn, this need for testing and implementation increased awareness of the importance of practice-based innovation structures [1] to strengthen primary care’s capacity to evolve as a learning organisation, thereby facilitating the implementation of the living lab. This perspective aligns with that of Vinnova, Sweden’s innovation agency, which has emphasised the growing importance of test arenas as services and technologies develop more rapidly and become increasingly complex [43]. In this context, a clinically based innovation and research infrastructure may further strengthen primary care’s role as a development-driven workplace by supporting competence building and attracting and retaining development-oriented staff. Moreover, industry–healthcare collaboration can facilitate the development of context-adapted innovations, while piloting solutions prior to procurement and wider implementation may improve efficiency and resource use. More broadly, systematic innovation work enhances organisations’ ability to create value, adapt to change and respond effectively to emerging challenges and opportunities [44]. Thus, the findings from this study may contribute to strengthening systematic innovation efforts in primary care organisations.

### Strengths and limitations

A key strength of this study is that it presents a complex process evaluation of a rarely studied phenomenon, offering insights for future implementation and contributing to organisational learning. Another notable strength is its grounding in extensive qualitative data collected over several years, including interviews across organisational levels and various documents, providing a rich and comprehensive picture of the implementation process. As this study focused on the implementation of a single living lab, generating quantitative data was challenging. Although process evaluations of complex interventions may benefit from both quantitative and qualitative data [21], purely qualitative approaches are often used [45]. Moreover, the credibility and reliability of the coding were strengthened by the fact that three of the authors (SS, E-LP and IS) were involved in the analysis, with E-LP offering a distinct outsider perspective. Two of the authors (IS and E-LP) had extensive experience with the method, having conducted several previous process evaluations. As a further strength, two of the authors (SS, IS) held insider positions, enabling an in-depth understanding of organisational processes and context, while their long-term involvement ensured sustained access and captured the implementation process over time.

This study also has some limitations. Although focus groups and dyadic interviews enabled interaction between participants during data generation, the analysis focused primarily on the verbal content of participants’ accounts rather than on interactional processes. Consequently, the study does not capture how meaning was co-constructed or negotiated between participants. Moreover, what participants chose to share during data collection may have been influenced by the fact that the first (SS) and last (IS) authors served as project leaders for the living lab. To reduce potential power asymmetry arising from SS’s role as a local project leader, SS did not conduct FG interviews with colleagues at the PCC concerning the living lab. These interviews were instead conducted by IS, who was less directly involved in the local context, together with researchers with an outsider perspective (E-LP and DH). However, two colleagues were interviewed (dyadic interview 1) by SS and IS after being informed in advance and offered an alternative, since they preferred this arrangement. To mitigate influence on participant responses, participants were explicitly encouraged during each interview to share both positive and negative experiences. Moreover, probing questions were used to counter superficial responses due to the researchers’ insider position, encouraging participants to elaborate and clarify assumptions [46]. In addition, the authors’ active involvement in implementing the living lab may have introduced analytical bias; this was addressed through a manifest, descriptive analysis [29], researcher triangulation (including both insider and outsider perspectives) and ongoing reflexivity [30]. Another limitation is that the patient/citizen perspective was not strongly represented. This was partly due to regulatory constraints that limited patient/citizen involvement in many living lab projects, restricting the opportunity to identify patient/citizen informants who could provide in-depth insights from their perspective.

Moreover, this implementation was conducted at a single PCC with the aim of informing future living lab implementations within the Swedish primary care context. We acknowledge that some findings may not be fully transferable across all primary care settings in Sweden or internationally, given contextual and regulatory differences. However, several underlying principles remain broadly applicable across primary care and other healthcare systems, including the need for practice-based arenas – as well as supportive leadership and strong employee engagement – to support innovation collaboration. Moreover, despite variation in local contexts and regulatory frameworks, the shared systemic pressures and evolving demands in healthcare, particularly the need for digital transformation and innovation, create a broadly comparable context.

## Conclusion and future research

The living lab emerged as a bottom-up initiative rooted in daily healthcare practice, continuously evolving in response to its environment. In this sense, the implementation process resembled the metaphor of ‘building the bridge as you cross it’. The findings indicate a broad interest in living labs in primary care, as they offer an opportunity for primary care practice to drive its own development. The living lab’s embeddedness made the flexibility and adaptability required for implementation possible within the dynamic system of primary care. At the same time, it introduced vulnerabilities that threatened its sustainability, as internal and external factors influenced the PCC’s capacity for innovation.

As this study focused on the implementation of a single living lab at a Swedish PCC, further research across diverse primary care contexts is needed to build a broader and more comprehensive evidence base and deepen understanding of living lab implementation, both in Sweden and internationally. Mixed-methods approaches may provide valuable insights into the effects of living labs on care quality, staff engagement and patient outcomes, while illuminating issues related to governance, digitalisation and patient participation.

## Supplementary Material

260429_Revised_Supplementary_File_1_CL.docx

Supplementary File 2.docx

## Data Availability

The datasets used and/or analysed during the study are available from the corresponding author upon reasonable request.

## Patient and public involvement

Patients and members of the public were not involved in this study.
