# Supplementary material for: Building the bridge as you cross it – a process evaluation of the implementation of a clinically integrated living lab in Swedish public primary care
Source: Scand J Prim Health Care. 2026 Oct 28;44(1):2752760. doi: 10.1080/02813432.2026.2752760 (PMC13721824; doi:10.1080/02813432.2026.2752760)
Supplement: Supplementary file 1 [file IPRI_A_2752760_SM7842.docx]

**Supplementary File 2**

**Focus group (FG) interview guides**

**FG 1: Executive managers in the fields of innovation, research and development**

- What is your job title, and what are your main responsibilities within VGR/the primary care organisation?
- Can you briefly tell us about your role in the development of the living lab at the primary care centre?
- How does the living lab fit into the region’s overall strategy for innovation and development in healthcare?
- What opportunities do you see with the living lab at the primary care centre?
- How do you assess the living lab’s relevance for
  1. The region’s strategy for the transition to high-quality, patient-centred care delivered close to home?
  2. The ongoing digitalisation in primary care?
  3. Promoting innovation within primary care?
- What do you see as the main challenges in implementing and managing a living lab in primary care?
- What are the most significant insights and experiences gained so far?
- How can the lessons and insights from the living lab be used and shared with other parts of healthcare?
- What are your thoughts on the future of the living lab?

**FG 2: Collaboration partners and innovation-supporting functions**

- Can you describe your involvement in the development and implementation of the living lab in primary care?
- Based on your different areas of expertise, what opportunities and challenges do you see in implementing a living lab within primary care?
- Do you think there is a need for living labs in primary care? If yes, in what way?
- What have you learned from being part of the living lab so far?
- What are your thoughts on the future of the living lab? What support do you think will be necessary going forward?

**FG 3: Primary care managers and research coordinator**

- Could you briefly tell us about your role in the development of the living lab at the primary care centre?
- Do you think there is a need for living labs in primary care? If yes, in what way?
- What opportunities and challenges exist or existed during the development of the living lab?
- What knowledge and skills are needed to run a living lab?
- What insights have you gained during the implementation of the living lab?
- Do you see a future for the living lab at the primary care centre? Please share your thoughts.

**FGs 4–5: Employees**

- What’s it like working with the living lab?
- Do you think there is a need for living labs in primary care? If yes, in what way?
- What has your experience been of implementing a living lab at the health centre?
- What factors are important for running a living lab at a primary care centre?
- In what ways has the living lab affected day-to-day work at the health centre?
- In what ways have you contributed to the different projects you’ve been part of?
- What’s it like working with internal projects (the region or primary care organisation) compared with projects with external partners (companies)?
- In what ways do you think the living lab affects the primary care centre’s ability to attract and retain staff?
- What do you think the future holds for the living lab?

**Final questions at each interview**

- Has anything we talked about today been unclear to you?
- Do you have any other thoughts or comments you would like to share?
