# Supplementary material for: Building the bridge as you cross it – a process evaluation of the implementation of a clinically integrated living lab in Swedish public primary care
Source: Scand J Prim Health Care. 2026 Oct 28;44(1):2752760. doi: 10.1080/02813432.2026.2752760 (PMC13721824; doi:10.1080/02813432.2026.2752760)
Supplement: Supplementary file 2 [file IPRI_A_2752760_SM7843.docx]

**Supplementary File 1**

Examples of living lab projects

| Type of project | Project holder | Participants | Activity (outcome) | Scientific publication | |
| --- | --- | --- | --- | --- | --- |
| An idea for a digitally connected toilet seat insert that enables automatic urine sampling, with results sent directly to the medical record | Start-up company (external) | 2 entrepreneurs,  1 nurse,  2 assistant nurses,  1 resident physician,  1 local project leader | To better understand urine-sampling practices in primary care, a focus group interview was conducted with healthcare professionals involved in urine sample handling.  (Entrepreneurs gained a better understanding of urine-sampling practices, and healthcare professionals emphasised the need for solutions that meet hygiene requirements and allow urine to be retained for culture.) | | No |
| Co-creation of an information video on fall prevention | Small company (external) | 2 entrepreneurs,  1 nurse,  1 occupational therapist (municipal rehab),  1 local project leader,  2 older people | An information video on fall prevention was co-created, to be displayed in waiting rooms at primary care centres and other public spaces in the community.  (The informational video has been shared and used in various settings, such as at meetings for older adults, and has been disseminated to regional and municipal healthcare.) | | [7] |
| A wound support application featuring a knowledge base on wound care and clinical decision support for healthcare professionals | Multinational company (external) | 1 IT director,  1 senior process application specialist,  1 general manager  Primary care centre:  1 nurse,  1 local project leader  Municipality:  12 nurses/district nurses,  1 medical secretary,  1 manager | The application was tested in clinical practice at the primary care centre and in home healthcare. Healthcare professionals provided feedback on usability and user experience during eight design meetings.  (The findings demonstrated the usefulness of digital tools in the assessment and treatment of chronic wounds and led to the initiation of a request for information (RFI) process as a step towards procurement.) | | [47] |
| Implementation of the regional patient contract guideline in practice | VGR (internal) | 2 representatives from VGR,  3 nurses,  1 resident physician,  1 psycho-therapist,  1 local project leader | The patient contract was tested and integrated into patient care workflows to assess its usability in practice.  (The patient contract was well received by staff and patients, but its implementation was hindered by the electronic health record system, which did not support documenting the process in a way that enabled its use in practice.) | | No |
| Implementation of a digital drop-in service with a chat function as an option for patients to access care | Public primary care organisation (internal) | 6 nurses/district nurses,  4 medical secretaries,  local project leader | The digital drop-in service was tested to develop effective workflows and identify facilitating and hindering factors in the implementation process.  (Benefits included learning and positive experiences; challenges were related to managing multiple systems, written communication and workload. Structured, stepwise training was suggested.) | | No |
| Development of a structured person-centred model for behavioural and psychological symptoms of dementia (BPSD) care in primary care settings | Employee-driven | 2 nurses specialised in dementia care,  project leaders | In the first step, an environmental scan will be conducted, including a literature review and focus group interviews. In the next step, a person-centred approach, including symptom assessment, will be developed, piloted and implemented.  (The project is ongoing, and no results are currently available.) | | Considered |
